# Post-fast refeeding enhances intestinal stem cell-mediated regeneration and tumourigenesis through mTORC1-dependent polyamine synthesis

**DOI:** 10.21203/rs.3.rs-2320717/v1

**Published:** 2023-01-10

**Authors:** Shinya Imada, Heaji Shin, Saleh Khawaled, Sven W. Meckelmann, Charles A. Whittaker, Renan Oliveira Corrêa, Dikshant Pradhan, Gizem Calibasi-Kocal, Luiza Martins Nascentes Melo, Gabriele Allies, Pia Wittenhofer, Oliver J. Schmitz, Jatin Roper, Marco Aurelio Ramirez Vinolo, Chia-Wei Cheng, Alpaslan Tasdogan, Ömer H. Yilmaz

**Affiliations:** 1 Department of Biology, The David H. Koch Institute for Integrative Cancer Research at MIT, MIT, Cambridge, MA 02139, USA; 2 Applied Analytical Chemistry, University of Duisburg-Essen, 45141 Essen, Germany; 3 Barbara K. Ostrom (1978) Bioinformatics and Computing Core Facility, Swanson Biotechnology Center, Koch Institute at the MIT, Cambridge, MA 02139, USA; 4 Laboratory of Immunoinflammation, Department of Genetics, Evolution, Microbiology and Immunology, Institute of Biology, University of Campinas, Campinas, SP 13083-862, Brazil; 5 Department of Translational Oncology, Institute of Oncology, Dokuz Eylul University, Izmir-Turkey, Turkey; 6 Department of Dermatology, University Hospital Essen & German Cancer Consortium, Partner Site, Essen, 45147, Germany; 7 Division of Gastroenterology, Department of Medicine, Duke University, Durham, North Carolina, USA; Department of Pharmacology and Cancer Biology, Duke University, Durham, North Carolina, NC 27710, USA; 8 Columbia Stem Cell Initiative, Department of Genetics and Development, Columbia University Irving Medical Center, New York, NY 10032, USA; 9 Department of Biology, The David H. Koch Institute for Integrative Cancer Research at MIT, MIT, Cambridge, MA 02139, USA; Broad Institute of Harvard and MIT, Cambridge, MA 02142, USA; Department of Pathology, Massachusetts General Hospital and Harvard Medical School, Boston, MA 02114, USA

## Abstract

For more than a century, fasting regimens have improved health, lifespan, and tissue regeneration in diverse organisms, including humans. However, how fasting and post-fast refeeding impact adult stem cells and tumour formation has yet to be explored in depth. Here, we demonstrate that post-fast refeeding increases intestinal stem cell (ISC) proliferation and tumour formation: Post-fast refeeding augments the regenerative capacity of *Lgr*5^+^ intestinal stem cells (ISCs), and loss of the tumour suppressor *Apc* in ISCs under post-fast refeeding leads to a higher tumour incidence in the small intestine and colon than in the fasted or *ad libitum* (AL) fed states. This demonstrates that post-fast refeeding is a distinct state. Mechanistically, we discovered that robust induction of mTORC1 in post-fast-refed ISCs increases protein synthesis via polyamine metabolism to drive these changes, as inhibition of mTORC1, polyamine metabolite production, or protein synthesis abrogates the regenerative or tumourigenic effects of post-fast refeeding. Thus, fast-refeeding cycles must be carefully considered when planning diet-based strategies for regeneration without increasing cancer risk, as post-fast refeeding leads to a burst not only in stem cell-driven regeneration but also in tumourigenicity.

## Introduction

A critical question in regenerative medicine is whether long-term dietary strategies can promote tissue regeneration without increasing cancer risk. For over a century, fasting interventions, including short-term fasting, iterative fasting, and caloric restriction, have been reported to extend life-span and enhance tissue regeneration in diverse species^1–6^, but whether and how adult stem cells can modulate this organismal benefit, given their importance in tissue regeneration and tumour initiation^7^, has not been fully understood thus far. Recent studies indicate that fasting interventions enhance tissue regeneration or repair after injury through changes in adult stem cells^3,8–10^. Fasting interventions also have noted inhibitory effects on tumour growth^2,11,12^. Still, whether and how fasting itself, post-fast refeeding, or both contribute to tissue regeneration or cancer initiation/progression remains unclear. Here, we aim to dissect the effects of the fasting-refeeding cycle on stem cell-mediated regeneration and tumour initiation using the mammalian intestine as a model.

The rapidly renewing mammalian intestinal epithelium responds to diet-induced physiological cues to dynamically alter the intestinal composition and adaptation^13,14^. Important in this process are actively cycling *Lgr5*+ intestinal stem cells (ISCs) at the crypt base, which are supported by epithelial, stromal, and immune cell niches^14–17^. These ISCs are maintained by niche-derived growth factors and developmental signalling pathways, such as WNT, NOTCH, BMP inhibitors, and EGF^18^, but are also regulated by environmental factors, such as the microbiome and diet^1,9,19,20^. Host diet and nutrients affect intestinal composition through direct and indirect alteration of ISCs^7,9,10,19,21^. Our group has previously shown that acute 24-hour fasting directly improves ISC function through the activation of a fatty acid oxidation program^9^, whereas a long-term calorically restricted (CR, 40% reduced caloric intake) diet indirectly augments ISCs by modulating mTORC1 signalling in the Paneth cell niche^10,21^. Thus, ISCs and their niches integrate diverse cues to dynamically actuate intestinal adaptations, but how nutritional and metabolic cues orchestrate ISC function and tumourigenesis remains to be further understood. Emerging evidence of post-transcriptional and translation regulation of stem cell maintenance, activation, regeneration, and tumourigenicity in other stem cell models^22–25^prompted us to investigate the role of post-transcriptional regulation in orchestrating the post-fast refeeding response. Here, we demonstrate for the first time that the fasting-refeeding cycle augments ISC-mediated regeneration after injury and tumourigenicity by regulating a novel mTORC1-polyamine metabolite-protein translation axis.

## Post-fast refeeding augments intestinal stem and progenitor cell proliferation and function

**Figure 1:**
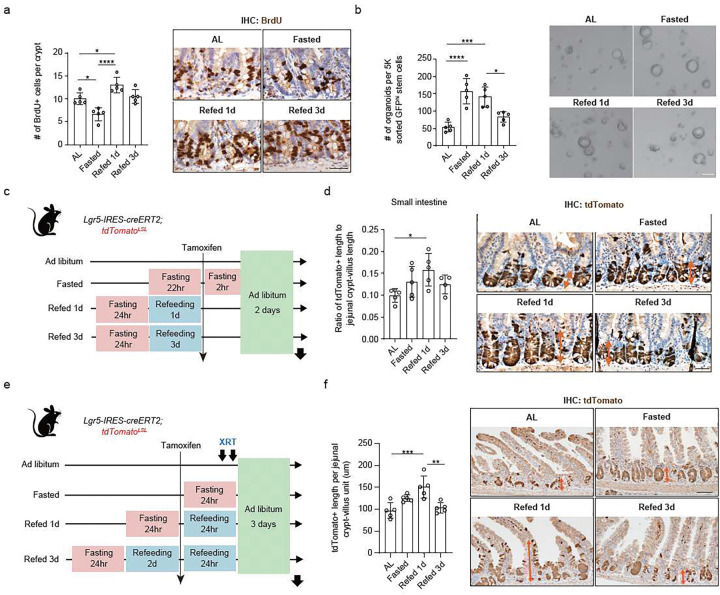
Post-fast refeeding augments intestinal stem and progenitor cell proliferation and function (a) Quantification (left) and representative images of BrdU+ cells (4 hours after BrdU administration) by IHC per jejunal crypt (right). n > 25 crypts per measurement, n = 5 mice per group. Scale bar, 25 μm. (b) Organoid-forming assay for FACS-sorted ISCs from AL, Fasted, Refed 1d, and Refed 3d mice. Quantification (left) and representative images of day 3 organoids (right). n = 5 mice per group. Scale bar, 50 μm. (c) Schematic of Lgr5 lineage tracing with Lgr5-IRES-CreERT2; Rosa26LSL-tdTomato reporter mice, including the timeline of tamoxifen injection and tissue collection. (d) Quantification (left) and representative images of tdTomato+ Lgr5+ ISC-derived progenies labeled by IHC for tdTomato (orange arrows, right) in the small intestine. n = 20 crypts per measurement, n > 4 mice per group. Scale bar, 25 μm. (e) Schematic of irradiation mouse model, including the timeline of irradiation (XRT 7.5Gy × 2) (f) Quantification (left) and representative images of IHC for tdTomato (right). n = 20 crypts per measurement, and n = 5 mice per group. Scale bar, 50 μm. Data in dot plots were expressed as mean ±SD. *p < 0.05, **p < 0.01, ***p < 0.001, ****p < 0.0001, one-way ANOVA.

To characterize the effects of post-fast refeeding in the intestine, we examined the following dietary conditions: *ad libitum* (AL), mice had free access to food; fasted, mice were deprived of food for 24 hours (h); refed, mice had free access to food following a 24 h fast for either 1 day (Refed 1d) or 3 days (Refed 3d). While fasting decreased body mass by 10–15%, post-fast refeeding for 1d restored the body mass to baseline (Extended Data Fig. 1a). To assess the effect of a fasting-refeeding cycle on ISC proliferation, we quantified the number of S-phase (BrdU positive cells after a 4h pulse) and M-phase cells (phospho-histone 3 positive cells) within the intestinal crypt where stem and progenitor cells reside. As previously reported, a 24 h fast significantly decreased crypt cell proliferation^9^. Post-fast refeeding, however, increased the number of proliferating crypt cells when compared to AL state (Fig. 1a; Extended Data Fig. 1b). Neither fasting nor post-fast refeeding changed crypt depth or villus height (Extended Data Fig. 1c), ISC numbers (Extended Data Fig. 1d, e), apoptotic cell numbers (Extended Data Fig. 1f, g), and Paneth and goblet cells per crypt (Extended Data Fig. 1h, i), indicating that ISC and progenitor cell proliferation was the most noticeable difference observed during a fasting-refeeding cycle.

Next, to investigate the impact of fasting and refeeding on ISC function, we assayed the ability of intestinal crypts and isolated ISCs (using *Lgr5-EGFP-IRES-CreERT2* reporter mice^20^) from each dietary condition to form *ex vivo* intestinal organoids (i.e., clonogenicity). Fasting and 1 d post-fast refeeding enhanced the organoid-forming capacity of intestinal crypts and ISCs, which returned to the AL baseline after 3 d post-fast refeeding (Fig. 1b, Extended Data Fig. 1j). The similarity between fasted and post-fast-refed ISCs/crypts may reflect the fact that fasted ISCs/crypts are cultured in nutrient-rich media, thus mimicking *in vivo* post-fast feeding.

Next, we sought to understand the effect of fasting and refeeding on *in vivo* ISC activity using the *Lgr5* ISC lineage tracer model (*Lgr5-IRES-CreERT2; Rosa26 loxp-stop-loxp (LSL)-tdTomato*)^26^. In this model, tamoxifen administration activated tdTomato labelling in ISCs and their progeny. We then quantified the tdTomato positive length along the crypt-villus axis after 2 d of tamoxifen administration under fasting and refeeding regimens (Fig. 1c). We found that 1 d refeeding increases the tdTomato-positive length by 60% in the small intestine and 35% in the colon compared to that of the AL controls, indicating that post-fast refeeding boosted ISC-mediated epithelial proliferation (Fig. 1d, Extended Data Fig. 1k). We also assessed whether post-fast refeeding mediates ISC-driven repair after radiation-induced injury. Using the aforementioned *Lgr5* ISC lineage tracer model, we labeled ISCs 24 h prior to radiation exposure with tdTomato, subjected mice to fasting/refeeding regimens, and then tracked tdTomato-positive cells for 3 d (Fig. 1e). Like homeostasis, the 1 d post-fast refeeding group showed the greatest effect on ISC-mediated repair after radiation-induced injury (Fig. 1f). Overall, our data support the conclusion that many benefits of fasting occur after refeeding by stimulating ISCs to generate greater numbers of progeny in homeostasis and after injury.

## Refeeding activates mTORC1 signalling in ISCs

To better understand how nutrient-sensing signalling pathways mediate ISC adaptation in response to fasting and refeeding, we assessed how these fasting and refeeding influenced the insulin/PI3K and mTORC1 pathways, which are known to promote eukaryotic cell growth and metabolism in response to environmental inputs, including nutrients and growth factors^27^. We first confirmed that the fasting/refeeding cycle changes these signalling responses: A 24-h fast caused blood glucose levels to decline by 50% compared to the AL state, which returned to AL baseline 30 min after post-fast refeeding (Extended Data Fig. 2a). Elevations in blood glucose levels stimulate insulin secretion, which in turn activates intracellular PI3K-AKT signalling and downstream mTORC1 signalling ^28,27^. Indeed, in intestinal crypts, we observed increased levels of phosphorylated AKT (S473) protein, which is a marker for AKT activation downstream of insulin-PI3K signalling, and increased levels of phosphorylated 40S ribosomal protein S6 (pS6) and eukaryotic translation initiation factor 4E-binding protein (p4EBP1), which indicate that mTORC1 activity is also activated shortly after refeeding (Extended Data Fig. 2b). To determine whether changes in mTORC1 activity occur downstream of the insulin-PI3K pathway, we administered a dual inhibitor of the insulin receptor (IR) and IGF-1 receptor (IGF-1R), OSI-906, and a selective PI3K inhibitor, BKM120, prior to refeeding^29,30^. Intestinal crypt immunoblots confirmed that treatment with these inhibitors abrogated the induction of pAKT, pS6, and p4EBP1 by refeeding (Extended Data Fig. 2b), demonstrating that PI3K-AKT signalling is required to induce mTORC1 activation in the refed state.

**Figure 2:**
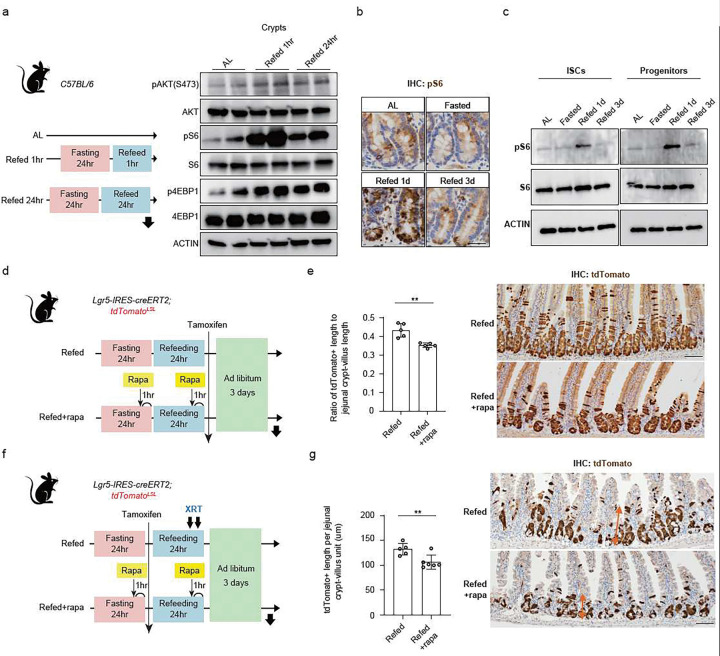
Refeeding activates mTORC1 signalling in ISCs (a) Schematic of the mouse model, including a refeeding period (left). Protein amount of phospho-AKT and mTORC1 downstream by immunoblots in crypts from AL, Refed 1h, Refed 24h mice (right). (b) Representative images of IHC for phospho-S6 in jejunal crypts from AL, Fasted, Refed 1d, and Refed 3d mice. Scale bar, 20 μm. (c) Protein amount of pS6, S6, and ACTIN in flow-sorted ISCs (Lgr5-GFPhi), progenitors (Lgr5-GFPlow) by immunoblots from each dietary conditioned Lgr5-IRES-creERT2 mice. (d) Schematic of homeostatic lineage tracing mouse model. (e) Quantification (left) and representative images of tdTomato+ Lgr5+ ISC-derived progenies labeled by IHC for tdTomato (right). Scale bar, 50 μm. (f) Schematic of the irradiation mouse model, including the timeline of rapamycin administration. (g) Quantification (left) and representative images of IHC for tdTomato (orange arrows, right). n=20 crypts per measurement, and n = 5 mice per group. Scale bar, 50 μm Data in dot plots were expressed as mean ±SD. **p < 0.01, Student’s t-test

We next measured mTORC1 activity in intestinal crypts and sorted ISCs and early progenitors at 1 or 3 days of refeeding. We identified that mTORC1 activity both at the early (refed 1 h) and the later refeeding (refed 1 d) time points were greater than that of the AL state (Fig. 2a). These changes were also noted at the crypt bottoms, including in Lgr5+ ISCs and their early daughter progenitors, as we saw increased pS6 levels by immunohistochemistry (IHC) and immunoblots in flow-sorted *Lgr5*-GFP^high^ ISCs and *Lgr5*-GFP^low^ progenitors (Fig. 2b–c). To determine whether elevated mTORC1 activity was required for refeeding-induced proliferation and regeneration, we administered the mTORC1 inhibitor rapamycin shortly before or during the refeeding phase. As expected, rapamycin treatment abrogated pS6 level in crypts (Extended Data Fig. 2c). Rapamycin treatment did not entirely block the refeeding-mediated hyperproliferation of crypt cells (Extended Data Fig. 2d); However, it did reduce the capacity of *Lgr5*+ ISCs to generate progeny in lineage tracing experiments (Fig. 2d, e) and their ability to regenerate the intestinal lining after irradiation-induced injury (Fig. 2f, g), as indicated by the decrease in tdTomato+ progenies derived from *Lgr5*+ ISCs. Thus, our data reveals that refeeding activates insulin-PI3K-mTORC1 signalling whereby mTORC1 activity boosts *Lgr5*+ ISCs activity during refeeding.

## Refeeding induces mTORC1-dependent transcriptional changes in stemness and metabolic pathways

**Figure 3:**
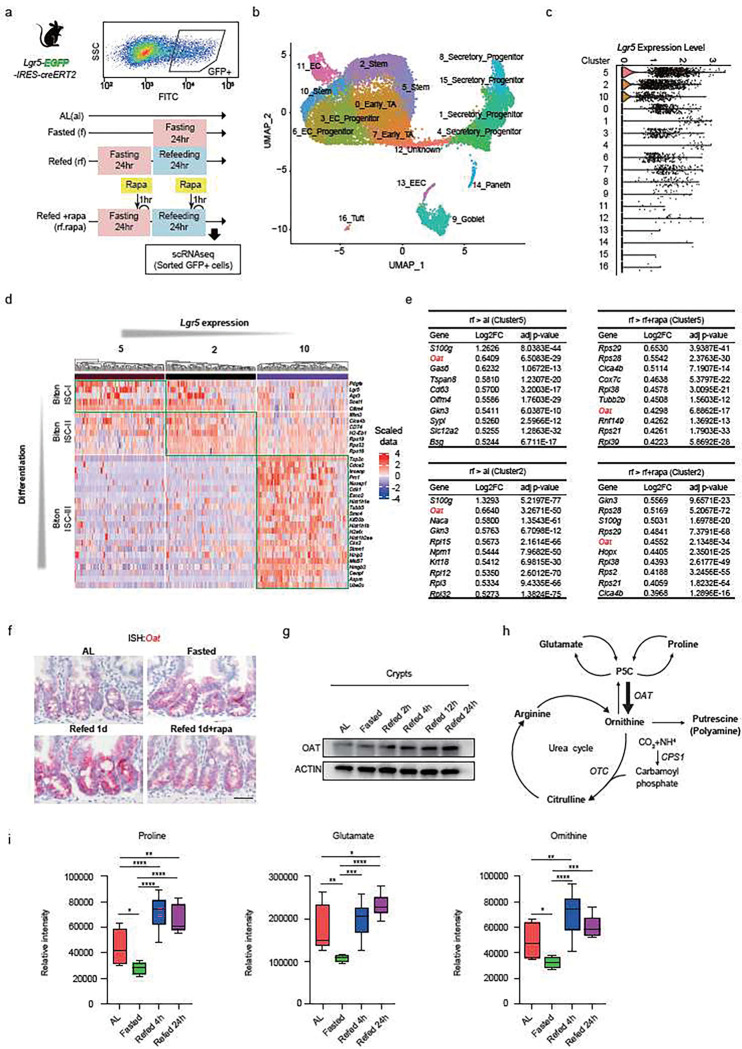
Refeeding induces mTORC1-dependent transcriptional changes in stemness and metabolic pathways (a) Schematic of single-cell RNAseq (scRNA-seq). GFP+ cells including ISCs (GFPhi) and progenitor cells (GFPlow) were flow-sorted from Lgr5-EGFP-IRES creERT2 mice. (b) Cell type clusters. UMAP for clustering (color coding) of 18,061 single cells (Ad libitum, n=1 and 4,760 cells; Fasted, n=1 and 4,282 cells; Refed 1d n=1 and 4,552 cells; Refed 1d with rapamycin treatment n=1 and 4,467 cells). TA, transit-amplifying (progenitor) cells; EC, enterocyte; EEC, enteroendocrine cells. (c) Lgr5 relative expression level among all clusters within all dietary groups. (d) Gene signatures comparison of ISC subsets between our stem cell clusters (5, 2,10) from all groups and Biton’s ISC classification. Representative genes of Biton’s ISC subsets are shown on the right side. (e) Top hit genes list in ISC subsets (5, 2) where refeeding (rf) stimulates these expression level compared to AL (al) or rf+rapa group. (f) Representative images of in situ hybridization (ISH, red) of OAT mRNA. Scar bar, 10 μm. (g) Immunoblots of OAT and ACTIN in the crypts from different timepoint. (h) Schematic of Ornithine metabolism including the metabolites and the genes encoding the catalytic enzyme. (i) The level of each metabolite with intestinal tissues from AL, Fasted, Refed 4h, and Refed 24h mice. n=4–5 per group. Data in dot plots were expressed as mean ±SD. *p < 0.05, **p < 0.01, ***p < 0.001, ****p < 0.0001, one-way ANOVA.

To gain greater insight in how mTORC1-mediated refeeding augments stemness, we performed droplet-based single-cell RNA sequencing (scRNAseq) analysis on FACS-sorted ISCs and their progenitor cells (GFP+) from *Lgr5-EGFP-IRES-CreERT2* reporter mice fed *AL*, fasted for 24 h (f), refed for 24 h (rf), and refed with rapamycin treatment (rf +rapa) (Fig. 3a). We performed UMAP analysis to partition GFP+ cells into 17 clusters based on the expression of established marker genes (Fig. 3b, Extended Data Fig. 3a)^31^, indicating that ISCs and progenitor cells are composed of various heterogeneous states. We identified three ISC clusters (clusters 5, 2, and 10) based on *Lgr5* expression levels (Fig. 3c), which closely matched previously identified ISC clusters by Biton and colleagues (termed Biton ISC-I, II, and III)^32^. Cluster 5 had the most stem-like and low-cycling features, similar to Biton-I. In contrast, cluster 10 had less primitive and proliferative characteristics mirroring Biton-III, and cluster 2 had intermediate features (Fig. 3d, Extended Data Fig. 3b, c).

Focusing on these three clusters, we first observed that their relative proportions did not change with fasting-refeeding (Extended Data Fig. 3d). Second, we addressed the proliferative state of the stem cell clusters. Consistent with our observations in intestinal crypts (Extended Data Fig. 2d), refeeding stimulated the proliferation of both clusters 5 and 2, which did not depend on mTORC1 activity (Extended Data Fig. 3e). Third, we performed gene set enrichment analysis (GSEA) of the three stem cell clusters (5,2,10) to evaluate the changes in stem cell signature gene expression, beginning with cluster 5 - the most primitive ISC subset (Fig. 3c, d). The distribution of the mean expression of Biton ISC-I gene signatures in cluster 5 ISCs revealed that refeeding strengthened the expression of these signatures; importantly, this induction was blocked by rapamycin treatment (Extended Data Fig. 3f). Like cluster 5, refeeding upregulated the Biton ISC-II gene signatures in cluster 2 through mTORC1 activation (Extended Data Fig. 3f). By contrast, neither fasting nor refeeding had positive effects on Biton ISC-III signatures in cluster 10 (Extended Data Fig. 3f). These data indicate that refeeding enhances the stemness program in the more primitive ISC subsets (5 and 2) in a mTORC1-dependent manner. We validated this enhancement using *in situ* hybridization (ISH) of the Biton ISC-I marker genes platelet-derived growth factor subunit A (*Pdgfa*) and gastrokine 3 (*Gkn3*), two genes elevated by refeeding in cluster 5 ISCs (Extended Data Fig. 3g, h).

To gain deeper mechanistic insights into the underlying refeeding-induced stemness program, we performed differential gene expression (DGE) analysis in ISC subsets, focusing on refeeding gene signatures that are regulated in an mTORC1-dependent manner. Ornithine aminotransferase (*OAT*) expression was one of the most upregulated genes in refed ISCs compared to AL state (rf > AL), and this induction was blocked by rapamycin treatment (rf > rf+rapa), especially in ISC clusters 5 and 2. (Fig 3e, Extended Fig. 3i). Consistent with DGE analysis, *Oat* mRNA transcripts in crypt base cells were strongly enhanced upon refeeding and dampened with rapamycin treatment (Fig. 3f). Protein analysis suggested a progressive increase in OAT proteins over 24 h of refeeding (Fig. 3g). *OAT* is a mitochondrial enzyme that is mainly expressed in the liver, intestine, brain, and kidneys. In the intestine, *OAT* converts ornithine from pyrroline-5-carboxylate (P5C) that is produced from proline and glutamate. Ornithine is a non-proteogenic amino acid used for either citrulline (urea cycle) or polyamine synthesis (Fig. 3h) ^33^. To understand how refeeding alters ornithine metabolism, we measured the levels of proline, glutamate, and ornithine in the intestinal tissue of mice that were subject to AL, fasted, refed for 4 h, and refed for 24 h. Notably, fasting decreased the levels of proline, glutamate, and ornithine in the intestine, while post-fast refeeding significantly increased their levels, starting from 4 h of feeding (Fig. 3i). Taken together, we uncovered that refeeding triggers mTORC1 dependent transcriptional changes in ISCs, and that post-fast refeeding boosts ornithine levels and metabolism (i.e., OAT upregulation during refeeding).

## Refeeding induces protein synthesis through mTORC1 and polyamine metabolism to drive regeneration

**Figure 4:**
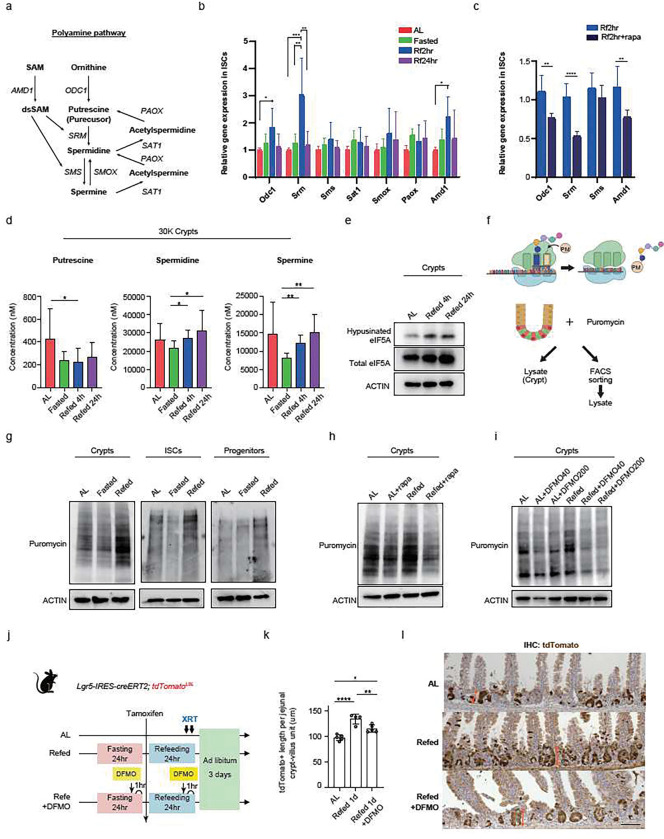
Refeeding induces protein synthesis through mTORC1 and polyamine metabolites to drive regeneration (a) Schematic of the polyamine biosynthesis pathway including the metabolites and the genes encoding the catalytic enzyme. (b) qPCR with FACS-sorted ISCs from AL, Fasted, Refed 2h, and Refed 24h mice. n=6–7 mice per group. (c) qPCR with FACS-sorted ISCs from Refed 2h and Refed 2h treated with rapamycin mice. n=6 mice per group. (d) Each polyamine level with intestinal crypt measured by LC-MS from AL, Fasted, Refed 4h, and Refed 24h mice. n=5 mice per group. (e) Protein levels of hypusinated eIF5A, Total eIF5A, and ACTIN in the crypt from different dietary conditioned mice. (f) Schematic of puromycin assay to address the protein synthesis (g) Immunoblots for puromycin and ACTIN in isolated crypts as well as in FACS-sorted ISCs (Lgr5-GFPhi),progenitors (Lgr5-GFPlow) labeled with puromycin from AL, Fasted, and Refed 1d Lgr5-EGFP-IRES-creERT2 mice. (h) Immunoblots for puromycin and ACTIN in isolated crypts labeled with puromycin from AL and Refed with or without rapamycin treatment. (i) Immunoblots for puromycin in crypts from AL and Refed with or without ODC1 inhibitor (DFMO). DFMO 40, 200: DFMO 40mg/kg, 200mg/kg. (j)(k)(l) Schematic of irradiation mouse model (j), quantification (k), and representative images of tdTomato+ Lgr5+ ISC-derived progenies labeled by IHC (l) (orange arrows). Scale bar, 50 μm Data in dot plots were expressed as mean ±SD. *p < 0.05, **p < 0.01, ***p < 0.001, ****p < 0.0001, one-way ANOVA for (b), (c), (k). The unpaired t test for (d).

Incorporation into the urea cycle and polyamine synthesis are two possible downstream metabolic fates for ornithine (Fig. 3h). In the urea cycle, carbamoyl phosphate synthetase 1 (*CPS1*) and ornithine transcarbamoylase (*OTC*) are key enzymes that catalyze citrulline formation from carbamoyl phosphate and ornithine (Fig. 3h). Given that *Cps1* mRNA levels were decreased in the early refed state (2 h) and that *Otc* mRNA levels were unchanged during refeeding (Extended Data Fig. 4a), we focused on the possibility that ornithine might be used for polyamine (putrescine, spermidine, and spermine) biosynthesis in ISCs and progenitors. Ornithine decarboxylase 1 (*ODC1*), the rate-limiting enzyme for polyamine production, catalyzes the conversion of ornithine into putrescine. Adenosylmethionine decarboxylase 1 (*AMD1*) is the second rate-limiting enzyme, which donates an amino-propyl group from S-adenosylmethionine for polyamine synthesis (Fig. 4a). First, we sought to address the expression levels of polyamine synthesis-related genes in sorted ISCs from *AL*, fasted, refed 2 h, and refed 24 h mice. We observed that both *Odc1* and *Amd1,* as well as spermidine synthase (*Srm*) mRNA levels were significantly upregulated in ISCs from refed 2 h mice compared to AL (Fig. 4b). Rapamycin treatment blunted the expression of these genes during refeeding, indicating mTORC1 dependent regulation of polyamine synthesis genes (Fig. 4c). Second, we quantitatively measured the polyamine levels in intestinal crypts from AL, fasted, and refed mice. These analyses revealed that the levels of spermidine and spermine decreased after fasting and increased in both refed 4 h and 24 h intestinal crypts, comparable to AL levels (Fig. 4d). Third, we assessed the level of eukaryotic translation initiation factor 5A (eIF5A), which is post-translationally modified by spermidine to generate hypusinated eIF5A and serves as a proxy for spermidine activity^34,35^. Hypusinated eIF5A amounts in both refed 4 h and 24 h crypts were greater than that of AL crypts (Fig. 4e), and increased hypusination of eIF5A in the refed state was suppressed by difluoromethylornithine (DFMO) treatment, an irreversible inhibitor of ODC^36,37^ (Extended Data Fig. 4b). We observed that ODC inhibition partially suppressed refeeding-activated mTORC1 signalling, indicating feedback regulation and crosstalk between mTORC1 signalling and polyamine synthesis, as previously noted in other mammalian tissues (Extended Data Fig. 4c)^38,39^.

Because polyamines and mTORC1 signalling regulate the global translation rate^27,40^, we measured protein synthesis rates in the different fasting/refeeding states using puromycin, a tyrosyl tRNA mimic that can be incorporated into nascent polypeptide chains to enable bulk protein measurement of protein synthesis ^21,24,25^ (Fig. 4f). Refed crypt cells, ISCs, and progenitor cells showed heightened protein synthesis rates with puromycin immunoblots compared to the other groups (Fig. 4g). Moreover, refeeding-induced protein synthesis was dampened by treatment with either mTORC1 or ODC inhibitors (Fig. 4h, i), indicating that activated mTORC1 signalling and increased polyamine levels enhance the global translation rate during refeeding. We next investigated whether increased polyamine levels contribute to refeeding-enhanced intestinal repair after irradiation (Fig. 1e, f): ODC inhibitor treatment blocked the enhanced ISC-mediated repair seen at the refed state after irradiation-induced injury (i.e. length of tdTomato+ ISC-derived progeny, Fig. 4j–l), indicating that the polyamines mediate refeeding-induced ISC activity. Our data indicate that refeeding through a mTORC1-polyamine-dependent manner stimulates global protein translation, thereby augmenting ISC-mediated repair after injury.

## Post-fast refeeding augments the tumourigenic capacity of ISCs

**Figure 5:**
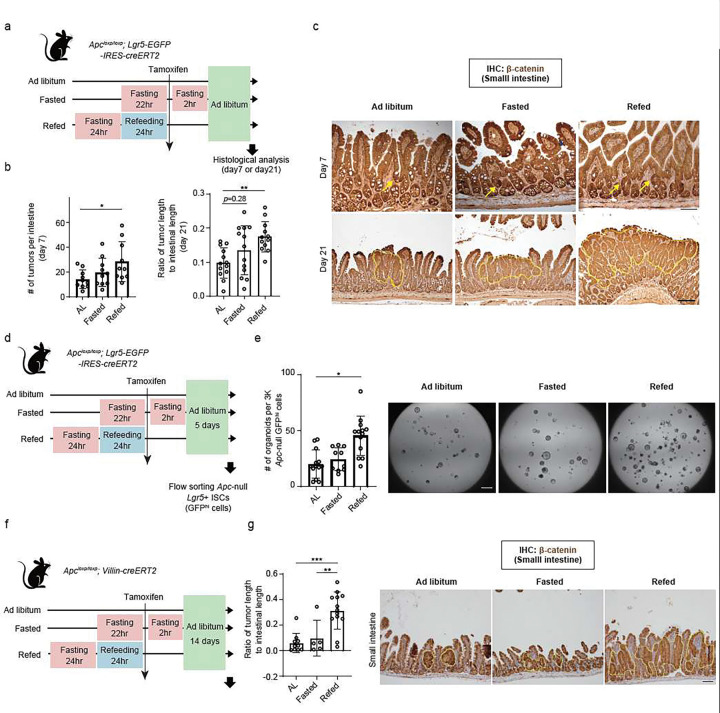
Post-fast refeeding augments the tumourigenic capacity of ISCs (a) Schematic of Apc tumour model with Apcloxp/loxp; Lgr5-EGFP-IRES-creERT2 mice. (b) The number of β-catenin+ nucleus Apc-null lesions 1 week after tamoxifen administration (left), and the ratio of tumour length in small intestine 3 weeks after tamoxifen administration (right). (c) Representative Apc-null tumour lesion by IHC for β-catenin. Tumours are pointed by yellow arrow or surrounded by a yellow dotted line. Scale bar, 100 m (upper) and 50 μm (lower). (d) Schematic of ex vivo adenomatous organoid model with FACS-sorted Apc-null ISCs (Lgr5-GFPhi) from Apcloxp/loxp; Lgr5-EGFP-IRES-CreERT2 mice. (e) Quantification (left) and representative day 6 images of Apc-null adenomatous (right). Scale bar, 1 mm. n = 5 mice (f) Schematic of Apc tumour model with Apcloxp/loxp;Villin-CreERT2 mice. (g) Ratio of β-catenin+ Apc-null tumour length in small intestine (left), and representative Apcnull tumour lesion by IHC for β-catenin. Tumours are surrounded by a yellow dotted line. Scale bar, 50 μm. Data in dot plots were expressed as mean ±SD. *p < 0.05, **p < 0.01, ***p < 0.001, one-way ANOVA.

Recent studies have demonstrated that fasting interventions reduce intestinal tumourigenesis, yet it is unclear how the timing of mutagenesis with respect to fasting and refeeding cycles influences tumour initiation^4,41–43^. Given the emerging role of protein synthesis in controlling stem cell regeneration and tumourigenesis ^23–25^and that refeeding enhances a mTORC1-polyamine dependent protein translation in ISCs, we postulated that post-fast refeeding may also alter intestinal tumour formation. To test this possibility, we utilized an *Apc* model of tumourigenesis in which the *Apc* gene, a tumour suppressor and a negative regulator of the Wnt pathway, can be ablated with tamoxifen administration: *Apc* was deleted in AL, fasted for 24 h (Fasted), and post-fast refed (Refed) states specifically in *Lgr5*+ ISCs using the *Apc*^*loxp/loxp*^: *Lgr5-EGFP-IRES-CreERT2* mouse model with tamoxifen (Fig. 5a–c). Tumour burden was measured by the number and the length of nuclear β-catenin+ *Apc*-null tumours in the small intestine at 7 or 21 d and in the colon at 21 d after tamoxifen administration (Extended Data Fig. 5a). Surprisingly, refed mice had greater numbers of tumours in the small intestine and colon at both time points (Fig. 5b, c, Extended Data Fig. 5b). These results do not reflect differential CreERT2 activity across different fasting or refeeding regimens, as CreERT2 activity of a Rosa tdTomato reporter strain was independent of the fasting-refeeding cycle (Extended Data Fig. 5c, d, e).

Next, we investigated whether refeeding enhanced the tumourigenic potential of *Lgr5*+ ISC in a cell-autonomous manner in organoid assays. We isolated an equal number of *Apc*-null *Lgr5*+ ISCs from AL, fasted, and refed mice 5 d after tamoxifen administration and assessed their capacity to give rise to *Apc*-null adenomatous organoids (Fig. 5d). We found that refeeding resulted in a two-fold increase in the number of ISC-derived *Apc*-null organoids, indicating that refed *Apc*-null ISCs had elevated organoid-forming capacity (Fig. 5e). Since the *Lgr5* knock-in allele shows mosaic expression in the *Lgr5-EGFP-IRES-CreERT2* mouse model^44^, we also induced tumours using *the Apc*^*loxp/loxp*^: *Villin-CreERT2* mouse model, which results in *Apc loss* in all intestinal epithelial cells, including ISCs, with tamoxifen administration (Fig. 5f). Like the *Apc*^*loxp/loxp*^: *Lgr5-EGFP-IRES-CreERT2* mouse model, refeeding augmented small intestinal and colonic tumour formation (Fig. 5g, Extended Data Fig. 5f), highlighting that post-fast refeeding enhances intestinal tumourigenicity, including those derived from *Lgr5*+ ISCs.

## Refeeding enhances ISC tumourigenic potential through mTORC1 and polyamine mediated protein synthesis

**Figure 6:**
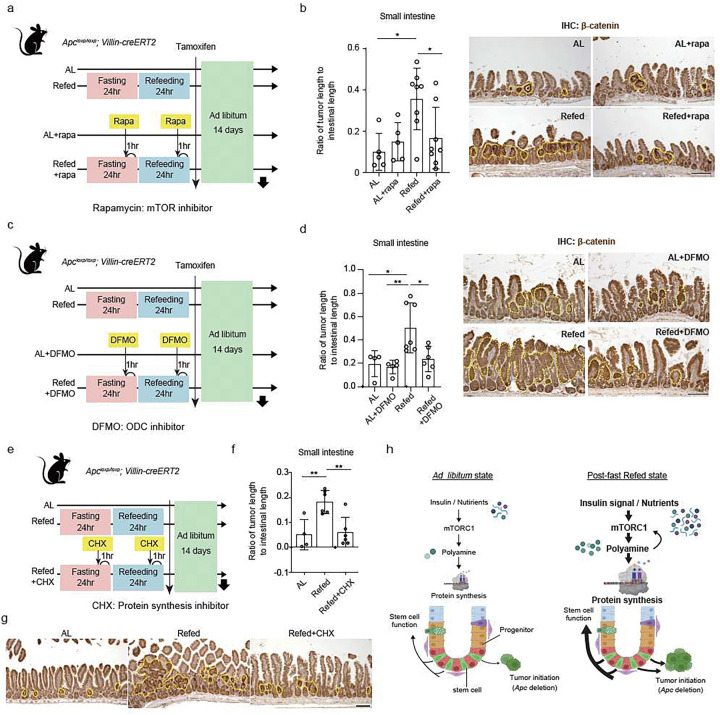
Refeeding enhances ISC tumourigenic potential through mTORC1 and polyamine mediated protein synthesis (a) (c) (e) Schematic assessing the effect of mTORC inhibitor (rapamycin) (a), ODC1 inhibitor (DFMO) (c), and protein synthesis inhibitor (cycloheximide) (e) on tumourigenesis using Apcloxp/loxp;Vllin- CreERT2 mouse model. (b) (d) (f) Quantification of tumour burden in the small intestine (left) and representative tumour images by IHC for β-catenin (right in b and d). Tumours are surrounded by yellow dotted lines. Scar bar, 100 μm. (b; rapamycin, d; DFMO). (g) Representative tumour images by IHC for β-catenin in the experiment of cycloheximide. (h) Model of how post-fast refeeding alters Lgr5+ ISC activity. Post-fast refeeding augments not only regenerative activity but also the intestinal tumourigenic capacity of Lgr5+ ISCs through the mTORC1 and spermidine mediated polyamine synthesis. Data in dot plots were expressed as mean ±SD. *p < 0.05, **p < 0.01, one-way ANOVA.

Although in established *Apc*-null intestinal tumours mTORC1 and ODC1 activity promote tumour progression^45,46^, the role of these pathways in early intestinal tumour formation in response to refeeding is unclear. To assess the link between mTORC1, refeeding, and tumour initiation in ISC, we administered rapamycin to AL and refed *Apc*^*loxp/loxp*^: *Villin-CreERT2* and *Apc*^*loxp/loxp*^; *Lgr5-EGFP-IRES-CreERT2* mice (Fig. 6a, Extended Data Fig. 6a). Notably, while rapamycin blocked the tumour initiating effects of refeeding in the small intestine and colon in both models, it did not affect tumour initiation effects in AL mice (Fig. 6b, Extended Data Fig. 6b, c). We then tested whether constitutive mTORC1 activity in ISCs akin to what occurs with refeeding, also boosts the tumourigenic capacity of *Lgr5*+ ISCs. We generated *Apc*^*loxp/loxp*^*: Tsc1*^*loxp/loxp*^; *Lgr5-EGFP-IRES-CreERT2* mice model, where *Tsc1*, a negative regulator of mTORC1, and *Apc* were disrupted in *Lgr5*+ ISCs with tamoxifen (Extended Data Fig. 6d). *Tsc1* co-deletion with *Apc* in ISCs resulted in a greater tumour burden (Extended Data Fig. 6e), supporting the notion that refeeding via mTORC1 stimulation promotes the tumourigenicity of *Lgr5*+ ISCs.

We also addressed the necessity of polyamines and protein synthesis in actuating the increased tumour-initiating capacity of the refed state. To inhibit polyamine production, we administered the ODC inhibitor DFMO to AL or refed *Apc*^*loxp/loxp*^: *Villin-CreERT2* mice (Fig. 6c) and found that ODC inhibition attenuated tumour formation in the refed intestine and colon with no effect on the AL cohort (Fig. 6d, Extended Data Fig. 6f). Also, given that mTORC1 and ODC inhibition blunted refeeding stimulated protein translation in intestinal progenitors (Fig. 4h, i), we treated refed *Apc*^*loxp/loxp*^: *Villin-CreERT2* mice with cycloheximide, an inhibitor of eukaryotic translation or protein synthesis, to ascertain whether dampening elevated protein translation in the refed state impacted intestinal tumour formation (Fig. 6e). Similar to rapamycin (Fig. 6a, b) and DMFO (Fig. 6c, d) treatment, a reduction in protein translation in the refed intestine (Extended Data Fig. 6g, h) suppressed the effects of post-fast refeeding on intestinal tumor formation (Fig. 6f, g). These finding demonstrate that post-fast refeeding by engaging an mTORC1- and polyamine-mediated protein synthesis axis elevates intestinal tumour initiation.

## Discussion

Recent studies have indicated that post-fast refeeding drives a unique cellular program distinct from fasting and AL feeding^47,48^. We and others have previously reported that a 24 h fast enhances ISC function by activating a fatty acid oxidation (FAO) program and that the fasted intestine and ISCs are protected from chemo- and radiation-induced damage^9,49^. The activation of FAO permits ISCs to survive low nutritional states by shifting energy metabolism towards lipids utilization (i.e., lipids that are released from adipose stores) and away from carbohydrate oxidation. Although activation of FAO maintains the ISC pool during fasting, we propose that post-fast refeeding strengthens the stemness attributes of ISCs. Firstly, after injury, the regenerative ability of refed ISCs exceeds that of the AL and fasted states (Fig. 1). Secondly, refeeding stimulates PI3K-AKT signalling and downstream mTORC1 signalling in ISCs, which persists for at least 1 d after refeeding (Fig. 2). Thirdly, post-fast refeeding stimulates global protein translation, which is coordinated, in part, by increased mTORC1 activity and polyamine metabolite synthesis (Fig. 6). Together, these findings support a model in which fasting primes (or permits the survival of) ISCs for a robust regenerative response upon nutritional stimulation (i.e., post-fast refeeding).

We also uncovered that polyamine metabolites mediate many aspects of post-fast refeeding enhanced intestinal stemness. Polyamines play an essential role in mammalian cell proliferation, differentiation, and apoptosis through diverse mechanisms, including protein and nucleic acid synthesis^50^. A recent study showed that polyamine levels regulate the translation rate in hair follicle stem cells (HFSCs), where altered translation rates affected stem cell fate decisions^51^. However, the role of polyamines in regulating stem cell adaptation to diet and tumour initiation is unknown. We identified that polyamine-induced protein translation in response to refeeding boosted the stemness program for ISC-derived regeneration. Finally, refeeding increased glutamate, proline, and ornithine levels, precursor metabolites that contribute to polyamine production. Future research will be needed to understand how refeeding-stimulated polyamine production alters stem cells in other tissues, whether polyamine rich diets emulate aspects of the post-fast refeeding response in the intestine and other tissues, and whether ornithine and elevated amino acids in refeeding have non-polyamine mediated effects on stem cell adaptation to refeeding.

An important implication of this study is the distinct effects that fasting and post-fast refeeding have on tumourigenesis. Previous studies have demonstrated that fasting interventions have mostly inhibitory effects on tumour growth^2,11,12^. However, these studies did not delineate the contributions of the fasted and refed states on tumour initiation. Our results suggest that post-fast refeeding can increase the risk of intestinal tumour incidence if mutagenesis occurs in the refed state relative to the AL or fasted states. Additional studies are needed to better understand how the proportion of fast-eat time, total calorie intake, and meal content during refeeding and whether repetitive cycles of fasting and refeeding, like intermittent fasting regimens, augment ISC function without increasing tumour incidence even when mutagenesis occurs during the refed part of the cycle. Collectively, our data indicate that post-fast refeeding leads to a burst in stem cell-driven regeneration and that these refed stem cells when exposed to genetic alterations have an elevated risk of spawning cancers; thus, careful consideration should be given to fast-refeeding cycles when planning diet-based strategies for regeneration without increasing cancer risk.

### CONTACT FOR REAGENT AND RESOURCE SHARING

Further information and requests for resources and reagents should be directed to and will be fulfilled by the Lead Contact, Ömer H. Yilmaz (ohyilmaz@mit.edu, (Ö.H.Y.)) or Shinya Imada (simada@mit.edu (SI)).

### EXPERIMENTAL MODEL AND SUBJECT DETAILS

#### Mice strain

Mice were under the husbandry care of the Department of Comparative Medicine in Koch Institute for Integrative Cancer Research. The following strains were obtained from the Jackson Laboratory: *Lgr5-EGFP-IRES-CreERT2* (strain name: B6.129P2-Lgr5^tm1(cre/ERT2)Cle^/J, stock number 008875), *Rosa26-lacZ* (strain name: B6.129S4- Gt(ROSA)26Sor^tm1Sor^/J, stock number 003474), *Rosa26LSL-tdTomato* strain (strain name: B6.Cg-Gt(ROSA)26Sor^tm9(CAG-tdTomato)Hze^/J, stock number 007909). *Villin-CreERT2* was a gift from Sylvie Robine and previously described^52^. For lineage tracing experiments, *Lgr5 -IRES-CreERT2*^26^(a gift from Dr. Hans Clevers) were crossed to *Rosa26LSL-tdTomato* strain. *Apc*^*loxp*^ exon 14 *(Apc*^*flox/flox*^) has been previously described^53^, and *Apc*^*loxp/loxp*^*; Lgr5-eGFP-IRES-CreERT2* were generated by crossing *Lgr5-eGFP-IRES-CreERT2*^44^ to *Apc*^*loxp/loxp*^ mice. *Apc*^*loxp/loxp*^*; Villin-CreERT2* mice were generated by crossing *Apc*^*loxp/loxp*^ mice to *Villin-CreERT2* mice^52^. In this study, both male and female mice were used at the ages of 2–4 months unless otherwise specified in the figure legends. *Tsc1*^*loxp/loxp*^ mice were the generous gift of D. Kwiatkowski (Harvard Medical School) and backcrossed to *C57BL/6* mice for at least 6 generations as previously reported ^10^, and *Apc*^*loxp/loxp*^*; Tsc1*^*loxp/loxp*^*; Lgr5-eGFP-IRES-CreERT2* were generated by crossing *Apc*^*loxp/loxp*^*; Lgr5-eGFP-IRES-CreERT2*^44^) to *Tsc1*^*loxp/loxp*^ mice. For the comparative experiment of Ad libitum, Fasted 24h, Refed 1d, and Refed 3d, fasting was achieved through food removal from mice at 10 AM and mice were sacrificed approximately 24 hr later. Post-fast refeeding for 1d was achieved through the return to *Ad libitum* at 10 AM after 24 hr fasting and mice were sacrificed approximately 24 hr later. Post-fast refeeding for 3d was achieved through the return to *Ad libitum* at 10 AM after 24 hours fasting and mice were sacrificed 72 hr later. Water was unlimited during fasting.

### METHOD DETAILS

#### In vivo treatments

Tamoxifen treatment: Tamoxifen injections were achieved by intraperitoneal (i.p.) injection suspended in sunflower seed oil (Spectrum S1929) at a concentration of 4 or 10 mg/ml, and intraperitoneally administered according to the time points indicated in figures and figure legends. Rapamycin treatment: Rapamycin (LC Laboratories) treatment was administered by intraperitoneal injection at 10 mg/kg at the time points indicated in figures and figure legends. As described previously ^10^, rapamycin was reconstituted in absolute ethanol at 10mg/ml and diluted in 5% Tween-80 (Sigma) and 5% PEG-400 (Hampton Research) before injection. The final volume of all injection was 150 μl. Cycloheximide treatment: Cycloheximide (Millipore sigma) was administered by intraperitoneal injection at 75 μl per 25g of body weight (15mg/kg) at the time points indicated in figures and figure legends. Cycloheximide was suspended in PBS at a concentration of 5 mg/ml. OSI-906 and BKM 120: Linsitinib (OSI-906, Med Chem Express) was suspended in 5% DMSO, 40% PEG-300, 5% Tween-80, and 50% ddH2O at a concentration of 6.25 mg/ml, then administered by oral gavage at 160 μl per 20g (50 mg/kg). Buparlisib (BKM120, Med Chem Express) was reconstituted in DMSO and diluted in sunflower seed oil (Spectrum S1929) at a concentration of 2.5 mg/ml, then administered by oral gavage at 200 μl per 20g (25 mg/kg). Both drugs were administered at the time points indicated in figures and figure legends. DFMO treatment: DFMO was reconstituted in PBS (10mg/ml) and administered by intraperitoneal injection at 40mg/kg or 200mg/kg. Irradiation experiments: Mice were challenged by a lethal dose of irradiation (7.5Gy × 2, 6 hours apart). Intestinal and colonic tissues were collected for histology 3 days after ionizing irradiation induced (XRT).

#### Crypt Isolation and culturing

As previously reported and briefly summarized here ^9^, small intestines were removed, washed with cold PBS, opened longitudinally and then incubated on the shaker machine at 4°C with PBS plus EDTA (10 mM) for 45 min. Tissues were then moved to PBS. Crypts were then mechanically separated from the connective tissue by shaking, and then filtered through a 70-μm mesh into a 50 mL conical tube to remove villus material and tissue fragments. Isolated crypts for cultures were counted and embedded in Matrigel (Corning 356231 growth factor reduced) at 8–10 crypts per μl and cultured in a modified form of medium as described previously ^10,54^. Unless otherwise noted, crypt culture media consists of Advanced DMEM (GIBCO) that was supplemented with EGF 40 ng ml^−1^ (PeproTech, 315–09), Noggin 200 ng ml^−1^ (PeproTech, 250–38), R-spondin 500 ng ml^−1^ (R&D, Sino Bioscience or ^55^), B27 1X (Life Technologies, 17504044), CHIR99021 3 μM (LC laboratories, C-6556), and Y-27632 dihydrochloride monohydrate 10 μM (Sigma-Aldrich, Y0503). 200–250 isolated intestinal crypts were re-suspended in 5 μl of above-mentioned media and 20 μl of Matrigel, and seeded in a flat bottom 48-well plate (Corning 3548). Matrigel is allowed to solidify for 20–30 minutes in a 37°C incubator. 300 μl of crypt culture medium was then overlaid onto the Matrigel, changed every three days, and maintained at 37°C in fully humidified chambers containing 5% CO2. Clonogenicity (colony-forming efficiency) was calculated by assessing organoid formation 3–6 days or as specified after initiation of cultures. If not directly used for cultures, crypts were then dissociated into single cells and sorted by flowcytometry ^10^. Flow isolated ISCs or progenitor cells were centrifuged at 200 g for 3 minutes, re-suspended in the appropriate volume of crypt medium and seeded onto 15 μl of Matrigel containing 1 μM JAG-1 protein (AnaSpec, AS-61298) in a flat bottom 48-well plate (Corning 3548). The Matrigel and cells were allowed to solidify before adding 300 μl of crypt culture medium.

#### qRT-PCR

25,000 single cells were flow-sorted into Tri Reagent (Life Technologies), and total RNA was purified according to the manufacture’s instructions with following modifications: the aqueous phase containing total RNA was purified using RNeasy plus kit (QIAGEN). RNA was converted to cDNA with cDNA synthesis kit (Bio-Rad). qRT-PCR was performed with diluted cDNA (1:5) in 2 wells for each primer and SYBR green master mix on Roche Light Cycler^®^ 480 detection system. The following primers are used for qRT-PCR: *Odc1* forward, 5’-GACGAGTTTGACTGCCACATC-3’; *Odc1* reverse, 5’-CGCAACATAGAACGCATCCTT-3’; *Srm* forward, 5’-CTTCCCCGTGGTGGACTAC-3’;*Srm* reverse, 5’-TGCTCGGGTTTTTGCTACACA-3’; *Sms* forward, 5’-CACAGCACGCTCGACTTCAA-3’; *Sms* reverse, 5’-TGCCATTCTTGTTCGTGTAAGTT-3’; *Amd1* forward, 5’- AGGGATCTGGGGATCTTCGTA-3’; *Amd1* reverse, 5’-TGCTTGTCAGTCTTTGTCACAC-3’; *Paox* forward, 5’-TGGGCTGGATTGCATCTTGG-3’; *Paox* reverse, 5’- AAAAGCGACCGTATCCTTGGG-3’; *Sat1* forward, 5’-GAGAACACCCCTTCTACCACT-3’; *Sat1* reverse, 5’-GCCTCTGTAATCACTCATCACGA-3’; *Smox* forward, 5’- TCCCACGGGAATCCTATCTATC-3’; *Smox* reverse, 5’-GCCACGGTTGGTAAGGTAGC-3’; *Cps1* forward, 5’-ACATGGTGACCAAGATTCCTCG-3’; *Cps1* reverse, 5’- TTCCTCAAAGGTGCGACCAAT-3’; *Otc* forward, 5’-AGGGTCACACTTCTGTGGTTC-3’; *Otc* reverse, 5’-CAGAGAGCCATAGCATGTACTG-3’; β*-Actin* forward, 5’- GGCTGTATTCCCCTCCATCG-3’; *β-Actin* reverse, 5’ CCAGTTGGTAACAATGCCATGT −3’.

#### In Situ Hybridization (ISH)

Single-molecule *in situ* hybridization was performed using Advanced Cell Diagnostics RNAscope 2.0 HD Detection Kit (Fast Red dye) for the following probes: Mm*-Lgr5* (Ref 312171), *Mm-Pdgfa* (Ref 411361), Mm*-Gkn3* (Ref 512061), Mm-*Oat* (Ref 545901)

#### Immunohistochemistry

As previously described ^20^, tissues were fixed in 10% formalin, paraffin embedded and sectioned in 5 micron sections. Antigen retrieval was performed with Borg Decloaker RTU solution (Biocare Medical) in a pressurized Decloaking Chamber (Biocare Medical) for 30 minutes at 95°C. Antibodies and respective dilutions used for immunohistochemistry are as follows: rat anti-BrdU (1:2000, Abcam 6326), rabbit monoclonal OLFM4 (1:10,000, CST #66479), rabbit polyclonal lysozyme (1:2000, Thermo RB-372-A1), rabbit monoclonal Cleaved Caspase-3 (1:500, CST #9664), rabbit polyclonal anti-RFP (1:500, Rockland 600–401-379), rabbit monoclonal phospho-H3 (1:500, CST #3377), rabbit monoclonal phospho-S6 ribosomal protein (1:500, CST #4858), mouse monoclonal β-catenin (clone14, 1:100, BD Transduction Laboratories). Biotin- conjugated secondary donkey anti-rabbit, anti-rat antibodies, or anti-mouse antibodies were used (1:500, Jackson ImmunoResearch). Vectastain ABC immunoperoxidase detection kit (Vector Laboratories PK-6101) was followed by Signalstain^®^ DAB subsrate kit for visualization (CST, 8049S). All antibody dilutions were performed in Signalstain^®^ Antibody Diluent (CST, 8112L).

#### Immunoblotting

The following antibodies were used for western blotting: Mouse monoclonal Actin (clone C4, 1:1000, Millipore sigma MAB1501), rabbit monoclonal S6 ribosomal protein (1:500, CST #2217), rabbit monoclonal phospho-S6 ribosomal protein (1:500, CST #4858), rabbit monoclonal 4E-BP1 (1:1000, CST #9644), rabbit monoclonal phospho-4E-BP1 (1:500, CST #2855), rabbit monoclonal phosphor-Akt (Ser473) (1:500, CST #4060), rabbit monoclonal Akt (pan) (1:1000, CST #4691), rabbit monoclonal Hypusine (1:1000, Millipore sigma #ABS1064), mouse monoclonal eIF5A (1:1000, BD Biosciences #611977), mouse monoclonal Puromycin (clone 12D10, 1:10,000, Millipore sigma MABE343). Isolated crypts were washed with cold PBS twice and eluted with RIPA buffer (CST #9806S). After centrifuging at 10,000 rpm for 15 minutes at 4°C, the resulting supernatant was calculated for protein concentration using Pierce^™^ BCA protein assay kit (Thermo Fisher Scientific 23225), then suspended in Laemmli SDS sample buffer (VWR J61337-AC) at a concentration of 1.5–2 ug/uμl, boiled for 5 min at 95°C. Samples were resolved by SDS–PAGE and analyzed by immunoblotting with horseradish peroxidase (HRP)-conjugated IgG secondary antibody (1:10,000, anti-rabbit CST #7074, anti-mouse CST #7076) and Western Bright Chemiluminescent detection kit (Advansta, ECL K-12045-D20, Sirius K-12043-D20). For the immunoblotting on flow-sorted cells, 2.5×10^4^ of each Lgr5-GFP^hi^ ISCs, Lgr5-GFP^low^ progenitors were sorted directly into 10 μl of Laemmli SDS sample buffer and boiled for 5 min. The samples were subjected to immunoblot analysis.

#### Protein synthesis assay

Protein synthesis was assessed using puromycin which incorporates at the C-terminus of nascent polypeptide chains. As previously reported^21^, isolated crypts were incubated at 37°C with minimum nutrition medium (Advanced DMEM supplemented with B27 1X and Y-27632 dihydrochloride monohydrate 10 μM) including 10 ug ml^−1^ puromycin for 15 min, centrifuged for 5 min, and then resulting pellet was eluted with RIPA buffer. The up-taken puromycin to the nascent polypeptide was analyzed with immunoblotting using mouse monoclonal anti-puromycin antibody (1:10,000, Millipore MABE343). For the protein synthesis assay on flow-sorted cells, isolated crypts from *Lgr5-EGFP-IRES-CreERT2* mice were incubated with 10 ug ml^−1^ puromycin, crypts were then dissociated into single cells and sorted by flowcytometry.

#### Blood Glucose test

1 drop of blood sample for each time point was obtained by cutting off the tip of tail (−1mm), and blood glucose was quickly measured using the Free Style Precision Neo System Kit and Blood Glucose Test Strip (Abbott).

#### The measurement of ornithine metabolism

##### Sample collection

3cm of intestinal tissue from the middle jejunum was minced for 1min using razor blades, and resuspended in cold PBS. Resuspended tissue was homogenized by a douncer, centrifuged for 2000g for 2 min to spin down tissues, then resulting supernatant was transferred to 1.5 ml tube. 200 μl of supernatant and 800 μl of 87.5% Ethanol preheated at 70 °C were incubated for 3 min, then 400 μl of supernatant was transferred following the centrifuge at 14000g for 3 min.

##### Flow-Injection TOF analysis for ornithine metabolomic

Non-targeted metabolomic experiment was conducted with General Metabolics (Boston, MA, USA). The analysis was performed on a platform on an Agilent 1260 Infinity II LC pump coupled to a Gerstel MPS autosampler (CTC Analytics, Zwingen, Switzerland) and an Agilent 6550 Series Quadrupole TOF mass spectrometer (Agilent, Santa Clara, CA, USA) with Dual AJS ESL source operating in negative mode as described previously^56^. The flow rate was 150 μl/min of mobile phase consisting of isopropanol : water (60:40, v/v) with 1 mM ammonium fluoride. For online mass axis correction, two ions in Agilent’s ESI-L Low Concentration Tuning Mix (G1969–85000) were used. Mass spectra were recorded in profile mode from m/z 50 to 1,050 with a frequency of 1.4s for 2 × 0.48 min (double injection) using the highest resolving power (4 GHz Hires).

##### Data Analysis for ornithine metabolomics

All steps of mass spectrometry data processing and analysis were performed with MATLAB (The Mathworks, Natick, MA, USA) using functions embedded in the Bioinformatics, Statistics, Database, and Parallel Computing toolboxes as described previously^56^. The resulting data included the intensity of each mass peak in each analyzed sample. Peak picking was done for each sample once on the total profile spectrum obtained by summing all single scans recorded over time, and using wavelet decomposition as provided by the Bioinformatic toolbox. In this procedure, a cutoff was applied to filter peaks of less than 5,000 ion counts (in the summed spectrum) to avoid the detection of features that are too low to deliver meaningful insights. Centroid lists from samples were then merged to a single matrix by binning the accurate centroid masses within the tolerance given by the instrument resolution. Starting from the HMDB v4.0 database (REF HMDB), we generated a list of expected ions including deprotonated, fluorinated, and all major adducts found under these conditions. All formulas matching the measured mass within a mass tolerance of 0.001 Da were enumerated. As this method does not employ chromatographic separation or in-depth MS2 characterization, it is not possible to distinguish between compounds with identical molecular formula. The confidence of annotation reflects Level 4 but-in practice-in the case of intermediates of primary metabolism it is higher because they are the most abundant metabolites in cells biological extracts. The resulting matrix lists the intensity of each mass of the values obtained from independent centroiding.

#### Sample collection and extraction for polyamine measurement

##### Chemicals

LC-MS grade acetonitrile, acetone and methanol were obtained from Avantor International (Darmstadt, Germany). LC-MS grade acetic acid, formic acid, and ammonium formate were purchased from Fisher Scientific (Schwerte, Germany). PBS (1x) was purchased from CLS (Eppelheim, Germany). Dansylchloride, spermidine, spermine, putrescine, N1-Ac-putrescine, N1-Ac-spermine, spermidine-^2^H_6_, spermine-butan-^2^H_8_ were from Sigma Aldrich (Steinheim, Germany). Ultrapure and desalted water with a resistivity of 18.2 M Ω/cm was generated by a Sartorius Stedim water purification system (Sartorius, Goettingen, Germany). All other chemicals were purchased by local distributor in the highest possible grade.

##### Extraction of Polyamines

Polyamines were extracted by a two-step liquid extraction adapted from Sellik et al. (2011 Nat Prot). Briefly, 30k crypt stem cells were snap frozen in liquid nitrogen for quenching. The frozen cells were extracted by adding 500 μL prechilled methanol (−80 °C), 20 μL of internal standard (arginine-^13^C_6_, spermidine-^2^H_6_, spermine-butan-^2^H_8_ at a concentration of 100 μM which will result in a concentration of 10 μM in the final extract) and vortexed for 2 minutes until no cell clumps were visible. Afterwards, the samples were sonicated for two minutes in a chilled (0 °C) ultrasonic bath. The cells were kept at −80 °C for 5 minutes and subsequently thawed. The thawed cells were vortexed and sonicated for 2 minutes and centrifuged at 3000 g for 5 minutes. The supernatant was collected and 250 μL of water acidified with 0.1 % acetic acid (LC-MS grade) was added. The samples were vortexed and sonicated for 2 minutes and subject to the same freezing-thawing cycle described before. The thaw samples were again vortex and sonicated for 2 minutes, respectively. Next, the samples were centrifuged at 3000g for 5 minutes and the supernatant is collected. The combined supernatant was dried in a vacuum-centrifuge for 45 min until complete dryness. The dried extract is resuspended in 200 μL acetonitrile/water (50/50; v/v) by sonicating and vortexing for 2 minutes, respectively.

##### Dansylation of Polyamines

Dansylation was carried out by using 80 μL of the obtained extracted of a standard solution with a known concentration. The solution was diluted with 80 μL of water, 40 μL of PBS (1x) and 20 μL of 1M NaOH. The reaction was started by adding 80 μL of a dansylchloride solution (10 mg/mL in acetone). The mixture was incubated at 55 °C for 30 minutes. Afterwards, the solution was dried in a vacuum centrifuge to complete dryness for 45 minutes. The residue was dissolved in 80 μL of acetonitrile/water (50/50; v/v) and subsequently analyzed by LC-MS.

##### LC-MS analysis and quantification

For polyamine analysis, an Agilent 1290 Infinity LC system coupled to an Agilent 6470 QqQ-MS was used (Agilent Technologies Inc., Waldbronn, Germany). Liquid chromatographic separation was carried out using a Zorbax Eclipse Plus C18 RRHD (50 × 2.1 mm, 1.8 μm; Agilent Technologies Inc., Waldbronn, Germany). The elution was carried out by utilizing a gradient at a flow rate of 400 μL/min with water (10 mM ammonium formate pH 3.5) as solvent A and acetonitrile/water (90/10; v/v; 10 mM ammonium formate pH 3.5) as solvent B. The linear gradient was: 0 min, 15% B; 2 min, 15% B; 7 min, 100% B; 10 min, 100% B followed by 2 min at initial condition for re-equilibration. Column temperature was 45 °C, injection volume 10 μL. Ionization was carried out in ESI positive mode by using the Agilent jet stream source. The following MS parameters were used: capillary 4500 V, nozzle voltage 1000 V, gas temp. 275 °C, gas flow 10 L/min, nebulizer gas pressure 25 psi, sheath gas temp. 350 °C, sheath gas flow 10 L/min. Detection was carried out in selected reaction monitoring (SRM) using the following optimized transitions for the transitions and parameters shown in table 1. Cycle time was 500 ms. Quantification of N1-Ac-putrescine, N1-Ac-spermine, putrescine, spermine and spermidine in crypt cells was performed by an external calibration in a range from 0.1 μM to 90 μM using 2 deuterated internal standards (spermidine-^2^H_6_ and spermine-butan-^2^H_8_) at a concentration of 10 μM. For calibration, the analyte to internal standard area ratios were linearly fitted to the corresponding concentration ratios and compared to the area ratios detected in the samples.

#### Droplet scRNAseq

Cells were sorted with the same parameters as described above for flow-cytometry into an Eppendorf tube containing 20 μl of minimum nutrition medium (Advanced DMEM supplemented with B27 1X and Y-27632 dihydrochloride monohydrate 10 μM) and stored on ice until proceeding to the Chromium Single Cell Platform. Single cells were processed through the Chromium Single Cell Platform using the Chromium Single Cell 3’ Library, Gel Bead and Chip Kits (10X Genomics, Pleasanton, CA), following the manufacturer’s protocol. Briefly, an input of 10,000 cells was added to each channel of a chip with a recovery rate of 4,000–4,500 cells. The cells were then partitioned into Gel Beads in Emulsion (GEMs) in the Chromium instrument, where cell lysis and barcoded reverse transcription of RNA occurred, followed by amplification, tagmentation and 5’ adaptor attachment. Libraries were sequenced on an Illumina NextSeq 500.

#### Droplet scRNAseq processing

The cellranger (version 3.1.0, 10x Genomics) application mkfastq was used to deconvolute raw fastq sequences and the application count was used to collapse UMIs, map to the mm10 mouse genome assembly and prepare count matrices. The cellranger filtered_feature_bc_matrix output data were imported into Seurat (version 3.2.0.)^57^ and genes detected in less than 3 cells per sample were excluded as were cells with fewer than 200 detected genes. No additional filtering was done based on mitochondrial gene expression. These criteria resulted in 4760, 4282, 4552 and 4467 cells for al, f, rf and rf.rapa respectively. The samples were merged into a single Seurat object and log normalized with a scaling factor of 10000. The vst method was used to identify the top 2000 most variable gene and dimensionality reduction was done using UMAP using 30 principal components. Initial examination of UMAP plots identified a subtle systemic difference between samples that were accounted for by dataset integration. Cell clusters were identified using a resolution value of 0.8 and clusters were assigned to cell type by examining the expression of individual genes or ssGSEA projections. Differential expression testing between clusters was done with the wilcox test implemented in the FindMarkers function. Pre-ranked GSEA was run using GSEA version 4.1^58^ using custom gene sets derived from Biton’s paper^32^ with log2 fold change from the differential expression testing as a ranking metric. To assess the cell cycle status in each cluster, the human g2m and s cell cycle gene lists provided by the Seurat package were mapped to mouse orthologs using Mouse Genome Informatics orthology data. The Percent Feature Set function was used to annotate each cell for the expression level of these sets. A Yes/No expression flag was then applied if percentage values were greater than 0.3 for the “g2m” set or 0.2 for the “s” set. The proportion of cells in each sample/cluster group was then tabulated for these flags. Single sample gene set enrichment analysis with Biton I/II/III gene set was performed using the escape version 1.0.0 running under R version 4.0.3. The R code used to process the single cell RNA-Seq data and prepare plots is available upon request.

#### Quantification and statistical analysis

Unless otherwise specified in the main text or figure legends, all sample numbers (n) represent biological replicates. Furthermore, organoid assays 3–5 wells per group at least 3 different mice were analyzed. The center values shown in box and whisker plots refer to the median while that in other graphs indicate mean. No sample or animals were excluded from analysis and sample size estimates were not used. Animals were randomly assigned to groups. Experiments used roughly equivalent male and female mice to avoid sex bias. Studies were not conducted blind with the exception of all histological analyses. Please note that statistical details are found in the figure legend. Statistical analysis was performed using GraphPad Prism 9. All experiments involving mice were carried out with approval from the Committee for Animal Care at MIT and under the supervision of the Department of Comparative Medicine at MIT.

## Figures and Tables

**Key Resource Table:** 

Reagents or Resource	Source	Identifier
Antibodies		
Rabbit RFP	Rockland	600-401-379
Rabbit phospho-H3	Cell Signaling Technologies	3377S
Rabbit phosopho-S6	Cell Signaling Technologies	4858S
Rabbit S6	Cell Signaling Technologies	2217S
Rabbit Olfm4	Cell Signaling Technologies	39141S
Mouse Actin	Millipore sigma	MAB1501
Mouse β-catenin	BD Biosciences	610164
Rat BrdU	Abcam	6326
Rabbit Lysozyme	Thermo Fisher	RB-372-A1
Rabbit phospho-4EBP1	Cell Signaling Technologies	2855S
Rabbit 4EBP1	Cell Signaling Technologies	9644S
Rabbit phospho-Akt (Ser473)	Cell Signaling Technologies	4060S
Rabbit Cleaved Caspase 3	Cell Signaling Technologies	9664S
Rabbit Akt (pan)	Cell Signaling Technologies	4691S
Mouse Puromycin	Millipore sigma	MABE343
Rabbit Hypusine	Millipore sigma	ABS1064
Mouse eIF5a	BD Biosciences	611977
CD45-PE, clone 30-F11	eBioscience	12-0451-83
CD31-PE, clone Mec 13.3	Biolegend	102514
Ter-119PE	Biolegend	116208
CD24-Pacfic Blue, clone M1/69	Biolegend	101820
CD117-APC/Cy7, clone 2B8	Biolegend	105826
EpCAM Apc, clone G8.8	eBioscience	17-5791-82
Chemicals, Peptides, and Recombinant Proteins		
PEG400	Sigma Aldrich	P4338
Tween-80	Sigma Aldrich	P4780
Tamoxifen	Sigma Aldrich	T5648-1G
Sunflower Seed Oil	Spectrum	S1929
Rapamycin	LC Laboratories	R-5000
Linsitinib (OSI-906)	Med Chem Express	HY-10191
Buparlisib (BKM120)	Med Chem Express	HY-70063
Cycloheximide	Millipore Sigma	C7698
Puromycin dihydrochloride	Millipore Sigma	P8833
DL-a-Difluoromethylornithine	Cayman	16889
Matrigel^™^	Corning	356231
Advanced DMEM	GIBCO	12491015
EGF	Peprotech	315-09
Noggin	Peprotech	250-38
R-spondin	Peprotech	315-32
*N*-acetyl-l-cystine	Sigma Aldrich	A9165
B27	Life Technologies	17504044
Chir99021	LC Laboratories	C-6556
Y-27632 dihydrochloride	Sigma Aldrich	Y0503
JAG-1	Anaspec	AS-61298
24 well plates	Olympus	25-107
48 well plates	Olympus	25-108
VECTASTAIN^®^Elite ABC kit, Peroxidase (Standard)	Vector Laboratories	PK6100
Signalstain^®^ DAB Substrate Kit	Cell Signaling Technologies	8049S
Signalstain^®^ Antibody Diluent	Cell Signaling Technologies	8112L
Western Bright Sirius	Advansta	K12043D20
Western Bright ECL	Advansta	K12045D20
CAPS BioXtra	Sigma Aldrich	C6070
Color Prestained Protein Standard, Broad Range	New England Biolabs	P7719S
TRI Reagent	Sigma Aldrich	93289
qScript cDNA Super Mix	Quantabio	95048
Perfect SYBR green fast mix	Quantabio	95072
Bovine Serum Albumin	Sigma Aldrich	A6003
Deposited Data		
RNA sequencing Data	GEO repository	GSE192482
Experimental Models: Organisms/Strains		
*Villin-CreERT2*	Dr. Sylvie Robine	N/A
*Lgr5-eGFP-IRES-CreERT2*	The Jackson Laboratory	008875
*Rosa26-LSL-lacZ*	The Jackson Laboratory	003474
*Apc^fl/fl^; Lgr5-eGFP-IRES-CreERT2*	Beyaz et al, 2016	https://pubmed.ncbi.nim.nih.gov /26935695/
*Lgr5-CreERT2*	Clevers Lab	https://www.ncbi.nlm.nih.gov/pmc/articles /PMC3634804/
*Lgr5-CreERT2; tdTomato^LSL^*	Cheng et al, 2019	https://pubmed.ncbi.nlm.nih.gov/ 31442404/
*Apc^fl/fl^; Villin-CreERT2*	Roper et al, 2017	N/A
Oligonucleotides		
See the part of Method Details for rt-PCR primers	Integrated DNA Technologies (IDT)	N/A
Software and Algorithms		
FlowJo v10	FlowJo LLC.	https://www.flowjo.com/
GraphPad Prism 8	GraphPad Software	https://www.graphpad.com/scientific-software/prism/
ImageJ-Fiji	National Institutes of Health, USA	https://fiji.sc/
Biorender	BioRender	https://app.biorender.com
Other		
RNAscope 2.0 HD Detection Kit	ACD RNAscope^®^	
RNA probe: *Mm-Lgr5*	ACD RNAscope^®^	Ref #312171
RNA probe: *Mm-Pdgfa*	ACD RNAscope^®^	Ref #411361
RNA probe: *Mm-Gkn3*	ACD RNAscope^®^	Ref #512061
RNA probe Mm-*Oat*	ACD RNAscope^®^	Ref #545901
